# Surface Modification of Copper-Based Flakes for Conductive Polymer Composites

**DOI:** 10.3390/polym16121620

**Published:** 2024-06-07

**Authors:** Mohor Mihelčič, Alen Oseli, Tadej Rojac, Lidija Slemenik Perše

**Affiliations:** 1 Faculty of Mechanical Engineering, University of Ljubljana, Aškerčeva Ulica 6, 1000 Ljubljana, Slovenia; mohor.mihelcic@fs.uni-lj.si (M.M.); alen.oseli@fs.uni-lj.si (A.O.); 2 Electronic Ceramics Department, Jozef Stefan Institute, Jamova Ulica 39, 1000 Ljubljana, Slovenia; tadej.rojac@ijs.si

**Keywords:** polymer composites, copper flakes, silver coating, silica coating, percolation threshold, rheological properties, thermal properties, mechanical properties, electrical properties

## Abstract

The physical properties as well as thermal and electrical stability of copper particles can be improved by surface protection, which mainly depends on the coating material. Our study was, therefore, focused on the rheological, thermal, mechanical and electrical characterization of polymer composites by comparing uncoated (Cu), silver-coated (Cu@Ag) and silica-coated (Cu@Si) copper flakes in low-density polyethylene at various volume concentrations (up to 40%). Interactions among particles were investigated by rheological properties, as these indicate network formation (geometrical entanglement), which is important for mechanical reinforcement as well as establishing an electric pathway (electrical percolation). The results showed that geometrical and electrical percolation were the same for Cu and Cu@Si, ~15%, while, surprisingly, Cu@Ag exhibited much lower percolation, ~7.5%, indicating the fusion of the Ag coating material, which also decreased crystal growth (degree of crystallinity). Furthermore, the magnitude of the rheological and mechanical response remained the same for all investigated materials, indicating that the coating materials do not provide any load transfer capabilities. However, they profoundly affect electron transfer, in that, Cu@Ag exhibited superior conductivity (74.4 S/m) compared to Cu (1.7 × 10^−4^ S/m) and Cu@Si (1.5 × 10^−10^ S/m). The results obtained are important for the design of advanced polymer composites for various applications, particularly in electronics where enhanced electrical conductivity is desired.

## 1. Introduction

The selection of a suitable filler is of great importance for the production and long-term behavior of various polymer composites, since the addition of fillers can provide various value-added properties, such as improving the mechanical, thermal, optical and electrical properties of the polymer material. As a result, the use of polymer composites has become ubiquitous in almost all areas of our daily lives.

Conductive Polymer Composites (CPCs) are composite materials consisting of an insulating polymer and conductive fillers that can conduct heat or electrical charge. They offer numerous advantages over conventional metal conductors and, therefore, they are gaining scientific interest in various research areas and applications such as sensors, actuators, electromagnetic interference shielding, heating elements, batteries and microelectronics [1,2,3,4,5]. The conductive fillers used in CPCs can be categorized into four types: metallic fillers (copper, silver, gold, etc.), carbon-based fillers (carbon black, graphene, CNT, etc.), ceramic fillers (BN, TiC, MXene, etc.) and metal-coated fillers (with or without a metal core) [6]. The insulating polymer matrix in CPCs offers flexibility, low cost, low density, corrosion resistance and high specific strength, as well as the production of complex shapes through various environmentally friendly technologies, such as 3D printing [7,8,9,10], injection molding [11] and compression molding [12].

Among the many conductive fillers, metal fillers are the most advantageous due to their high electrical conductivity, which can reach up to 10^7^ S/m. Silver (Ag), copper (Cu) and gold (Au) are the most conductive. Moreover, in addition to their high conductivity, Ag and Au are not prone to oxidation in air at elevated temperatures [13]; however, their high price is a major disadvantage. The most promising metal filler is undoubtedly Cu since it is an ideal conducting material for electronic applications due to its high electrical and thermal conductivity, low cost and low electron migration. Its main drawback is that it has an inherent tendency to oxidize at ambient conditions because of the relatively low Cu^0^/Cu^2+^ redox potential [14]; moreover, it fails to maintain high conductivity for a long period of time.

From a practical point of view, conductive metal fillers are usually the most expensive materials in conductive composites. Hence, to achieve the maximal efficiency (mechanical properties and conductivity) the amount of filler should be as high as possible; however, high concentrations of filler lead to difficulties in processing and increase the costs. Determination of the best ratio of the metal filler and polymer matrix is, therefore, extremely important to achieve optimal functional properties and economic efficiency.

Several strategies have been developed to improve the physical properties of Cu particles to obtain air-stable Cu particles. The most efficient way is to protect the surface of the particle by coating it with proper protective layers such as long-chain surfactants, organic polymers, inorganic materials, or carbon-based materials [9,15,16,17,18,19,20,21] in order to improve the conductivity. Ag is usually used as the best inorganic conductive element. Cu@Ag (core@shell) particles may be prepared either by an electroless type of substitution plating by the substitution reaction between Cu^0^ and Ag^+^ [17,22], thermal evaporation under ultrahigh vacuum [23], or ion exchange in a soda-lime glass matrix [24]. Ag-coated Cu powder is mainly used as a filler in electrically conductive adhesives (usually a thermosetting epoxy resin), where spherical particles are suitable for isotropic conductive adhesives, while flake particles are suitable for anisotropic conductive adhesives. Due to the presence of Ag, the oxidation of Ag-coated Cu powders decreases; however, the degree of oxidation directly depends on the Ag content on the surface of the Cu particles. It has been reported in the literature that Cu@Ag powders coated with 15 wt.% Ag resulted in stable electrical performance for about 2 h at 200 °C, but when temperatures rose above 250 °C, the electrical resistance started to increase rapidly. At the same time, it has been observed that compared to silver fillers, Ag-coated copper fillers (Cu@Ag) form more conductive composites, which means that filaments for 3D printing based on Cu@Ag fillers would be able to form conductive networks with higher conductivity than silver fillers [25].

Another way to prevent oxidation/degradation of the copper particle surface is to use metal oxides as shell materials around the core particles, such as SiO_2_, TiO_2_, or Al_2_O_3_, where silica is, due to its low-cost synthesis, the most promising for the production of core-shell particles. Silica has outstanding properties such as thermal stability, chemical inertness, optical transparency and susceptibility to surface modification. The shell around the particle is usually formed by a sol–gel reaction using silicon alkoxides as precursors in ethanol–ammonia mixtures, which allows the growth of a silica shell around the particles [26,27]. In contrast to silver, a silica coating forms an insulating layer that decreases the conductivity of the metal particle but improves the thermal conductivity.

In general, a polymer composite becomes conductive when the concentration of filler particles reaches a critical value, known as the percolation threshold, which indicates the formation of a conductive network within the non-conductive polymer. Once the percolation threshold is exceeded, the conductivity increases towards a plateau, approaching the conductivity of the filler. This phenomenon can be described by percolation theory [28]. In order to achieve high electrical conductivity in polymer composites, the shape and size of the filler and filler loadings are the most important factors in establishing the geometric and, consequently, conductive network. In addition, other factors, such as the dispersion of the filler, filler–polymer interactions, the size and distribution of the filler, the aspect ratio (*A_r_*) of the filler and the surface functionalization, also affect the formation of a conductive network in the composite [29]. Since fillers can occur in different morphologies, they have a significant influence on the final structure of the polymer composite and consequently on its final physical properties. In addition to filler properties, other factors also influence the final properties of the composite, such as the type of the polymer (polarity, surface tension, viscosity, degree of crystallinity, chain branching) and the content and type of additives and impurities, as well as the technology used in the production of the composites [12,30].

As Cu is one of the most important fillers in the field of CPCs, there are several publications in the literature regarding their use and surface modifications. Several possibilities are used to protect the Cu surface from oxidation, where Ag and Si coatings have proved to be very promising. Surface modification of conductive particles significantly affects the formation of a particle network and, consequently, the final properties, including the conductivity of CPCs, which is insufficiently addressed in the literature. Our study was, therefore, focused on the investigation of the effect of surface modifications of Cu flakes on network formation (geometrical and electrical) and consequently on reinforcing and conductive functionality of prepared composites. The study was performed with uncoated Cu (Cu), silver-coated Cu (Cu@Ag) and silica-coated Cu (Cu@Si) flakes in relation to different filler contents, i.e., from 0 to 40 vol.% in a low-density polyethylene matrix material.

## 2. Materials and Methods

### 2.1. Materials

Cu-based composites were prepared using commercially available low-density polyethylene-LDPE (780E NATURAL, Dow Chemical Company, Midland, MI, USA) with a density of *ρ_LDPE_* = 0.923 g/cm^3^ and copper-based fillers with different metal coatings: uncoated copper flakes, Cu (STANDART Lac L 900, Eckart, Hartenstein, Germany) with a density of *ρ_Cu_* = 8.5 g/cm^3^, silver coated copper flakes, Cu@Ag (eConduct Cu 341000, Eckart, Hartenstein, Germany) and silica-coated copper flakes, Cu@Si (STANDART RESIST LT, Eckart, Hartenstein, Germany). The mass concentration, *Φ_m_*, of coating material was constant, i.e., *Φ_m_* = 10% for Ag and Si.

**Figure 1:**
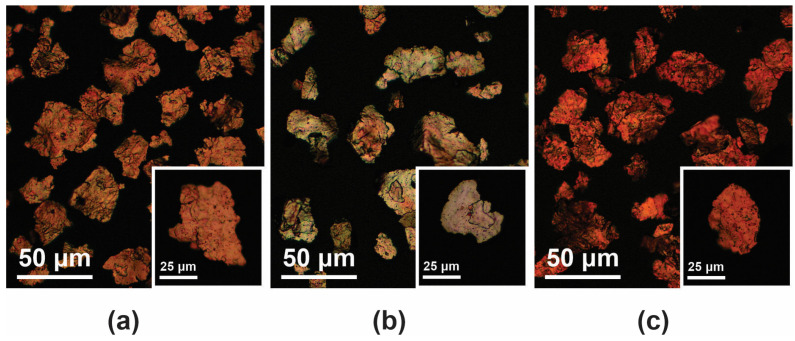
Optical micrographs for identification of geometry and surface microstructure of (**a**) Cu, (**b**) Cu@Ag and (**c**) Cu@Si flakes.

Preliminary optical micrographs were taken at 200× and 500× magnifications using an optical microscope (AxioScope 2, Carl Zeiss, Braunschweig, Germany) to determine geometry and surface microstructure of investigated Cu flakes (Figure 1). All three types of flakes used exhibited irregular flake edges and a coarse flake surface with an average equivalent diameter of ~30 µm and thickness of ~0.7 μm, giving an aspect ratio of ~40. Furthermore, the surfaces of the flakes were not uniformly covered with the coating material. In addition, the Cu@Ag flakes had a greyish appearance, while the Cu@Si flakes had a more brilliant copper appearance, as stated by the manufacturer.

### 2.2. Preparation of the Composites and Samples for Characterization

The Cu-based composites were prepared by melt processing at 190 °C in a twin-screw extruder (Xplore Micro compounder MC 15 HT, Xplore Instruments, Sittard, The Netherlands), using co-rotating mode with excellent mixing and dispersion capabilities. In order to achieve a homogeneous distribution of the filler within LDPE matrix material, a two-step homogenization process was applied. In the first step, 50% of the filler was added to matrix material and homogenized. The remaining amount of the filler was in the next step added to the mixture, which was then extruded and pelletized for final sample preparation. During the homogenization process, each step consisted of a dosing period of 5 min at a screw speed of 25 rpm and a mixing period of 10 min at a screw speed of 50 rpm at a temperature of 190 °C. Prior to the preparation process, LDPE granules were dried for 24 h at 60 °C under nitrogen atmosphere using a modified laboratory temperature chamber (SP-105C, Kambič, Slovenia), while nitrogen gas was also added to the system during the homogenization process to mitigate oxidation of Cu flakes. For the investigated fillers, several mixtures were prepared containing volume concentrations, *Φ_vol_*, of Cu flakes from 0 to 40%.

The samples for characterization purposes were prepared using the injection molding technique (Xplore micro injection molder IM 12, Xplore Instruments, Sittard, The Netherlands), in which pelletized extrudates were dosed into the injection molding piston, previously heated at 190 °C for 3 min, and then molded into a final shape using an injection pressure of 1000 bar for 10 s and a holding pressure of 150 bar for additional 10 s. The temperature of mold was set to 80 °C. For the highest volume concentrations (*Φ_vol_* = 40 vol.%), the mold temperature was increased to 105 °C to achieve complete filling of the tool.

**Figure 2:**
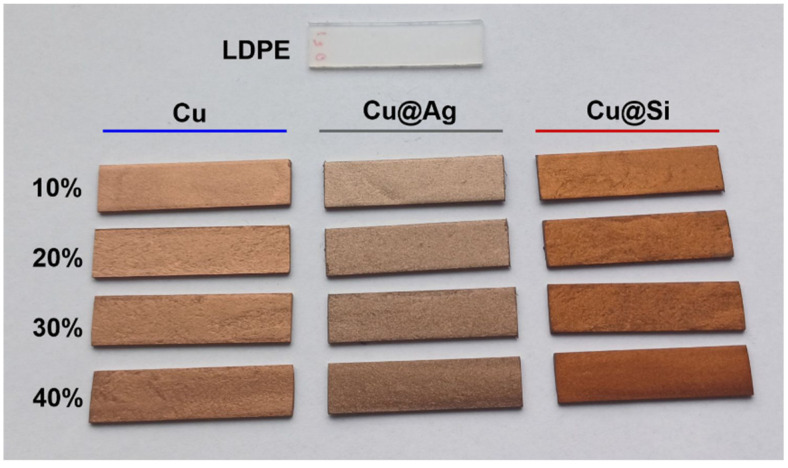
DMA samples of LDPE and Cu-based composites with different volume concentrations of fillers.

Two different sample geometries were prepared, i.e., disk-shaped samples for rheological characterization with a diameter of 25 mm and thickness of 1.5 mm, while for thermal, mechanical and electrical characterization, DMA test bars (Figure 2) were produced with a length of 60 mm, width of 10 mm and thickness of 1 mm.

### 2.3. Characterization Methods

#### 2.3.1. Rheological Analysis (Particle Interactions and Network Formation)

In order to identify particle interactions (indicating network formation), rheological analysis was performed using a rheometer (MCR302, Anton Paar, Graz, Austria). Tests were conducted at a constant temperature of 190 °C with plate–plate sensor geometry and a gap of 1 mm. For low volume concentrations, a sensor with a 25 mm diameter was used; while for high filler loadings, i.e., 30 and 40%, an 8 mm disposable sensor was selected due to the high consistency of the samples. Viscoelastic properties, i.e., storage modulus (*G*′) loss modulus (*G*″) and complex viscosity (η*), were determined by frequency sweep tests in the range of 100 Hz to 0.01 Hz within the linear viscoelastic domain (preliminarily determined by amplitude sweep tests), using 0.01% strain excitation for low and 0.05% for high volume concentrations. Extremely small excitation magnitudes were applied to avoid consistency and network changes due to applied load. Results of viscoelastic properties are presented as the average value of 3 repetitions for each investigated material, whereas error bars indicate maximum deviation from the average value.

#### 2.3.2. Thermal Analysis (Effect of Particle Interactions on Crystalline Structure)

To evaluate the effect of particle interactions on the structure of the matrix material, thermal analysis was conducted using differential scanning calorimetry, DSC (Q2500, TA Instruments, New Castle, DE, USA). The measurements were performed in the temperature range of 25 °C to 250 °C with a heating and cooling rate of 10 °C/min in inert nitrogen atmosphere with gas flow of 50 mL/min. Results of thermal properties, i.e., crystallization temperature (*T_c_*), melting temperature (*T_m_*) and degree of crystallinity (*X_c_*), are presented as the average value of 3 repetitions for each investigated material, whereas error bars indicate maximum deviation from the average value. For determination of *X_c_*, a fusion enthalpy Δ$H_{m}^{0}$ of 291.2 J/g was used for completely crystalline LDPE [31].

#### 2.3.3. Dynamic Mechanical Analysis (Effect of Particle Interactions on Mechanical Reinforcement)

The effect of particle interactions on the mechanical reinforcement of the Cu-based composites was evaluated through dynamic mechanical analysis using a rheological system (MCR702, Anton Paar, Graz, Austria). Tests were carried out in bending mode using a 3-point bending sensor geometry in the temperature range of 15 °C to 80 °C with a heating rate of 3 °C/min under inert nitrogen atmosphere. Furthermore, measurements were performed within the linear viscoelastic domain (preliminarily determined with amplitude sweep tests at 80 °C) using 0.1% strain excitation for low and 0.5% for high volume concentrations. Particularly small deformations were used to avoid structural damage of the established Cu network. Results of mechanical properties, i.e., storage (*E*′) and loss (*E*″) modulus, are presented as the average value of 3 repetitions for each investigated material, whereas error bars indicate maximum deviation from the average value.

#### 2.3.4. Electrical Analysis (Effect of Particle Interactions on Electrical Conductivity)

To investigate how particle interactions affect the conductive performance of selected composites, electrical analysis was conducted at room temperature using a custom-built 4-wire DC measuring rig coupled with a high voltage source measuring unit (Keithley 237, Keithley Instruments, Solon, OH, USA). By measuring a current for a given excitation voltage ranging from −120 V to 120 V, specific resistivity *ρ* and conductivity *σ* of investigated composites were determined. Results of *σ* are presented as the average value of 3 repetitions for each investigated material, while the error presents max. deviation from the average value.

## 3. Results and Discussion

### 3.1. Rheological Analysis (Particle–Particle Interactions and Network Formation)

**Figure 3:**
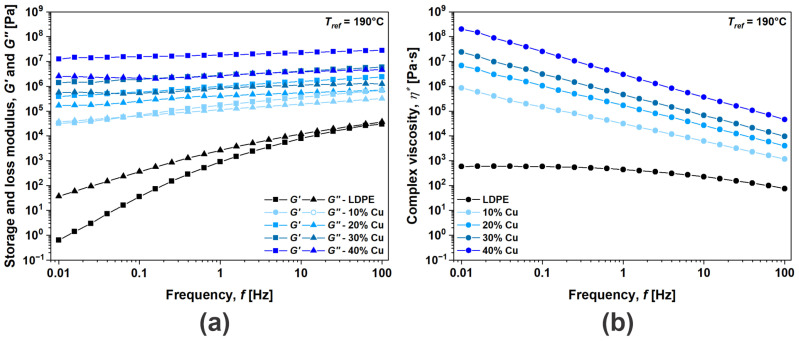
Viscoelastic properties: (**a**) storage and loss modulus; (**b**) complex viscosity as a function of frequency for Cu composites.

Rheological analysis presents one of the most important tools for investigating particle interactions and their impact on the performance (i.e., mechanical reinforcement, electrical conductivity) of functional composites [12,32,33]. This is especially important when considering Cu-based fillers, which are prone to oxidation and other thermo-chemical reactions at elevated temperatures. In order to determine time-dependent viscoelastic properties, i.e., storage modulus (*G*′), loss modulus (*G*″) and complex viscosity (η*), frequency tests were performed in the linear viscoelastic range (Figure 3). For convenience, only the data for Cu composites are presented, however, the same trends were observed for the other two filler materials investigated, i.e., Cu@Ag and Cu@Si. The frequency-dependent viscoelastic response was first determined for neat LDPE, as polymer relaxation dynamics determines the frequency range for evaluation of the complex particle interactions of the investigated composites. At high frequencies, molecules have limited time to disentangle and act as an entangled system, exhibiting a solid-like response in which storage modulus dominates (*G*′ > *G*″). As the frequency decreases, molecules have more time to react and they completely disentangle, displaying flow-like behavior in which loss modulus dominates (*G*″ > *G*′). This frequency region, also known as the terminal region with *G*″ > *G*′ and η* plateauing, was for LDPE determined in the frequency range from 0.01 Hz to 0.1 Hz. Within this region, where the material exhibits a flow-like response, complex interactions among particles may be determined. However, the transition from solid- to liquid-like response, usually determined with cross-over frequency ω*_co_*, was here found to be outside the experimental window, i.e., above 100 Hz. Rheological characterization of the Cu-flake-based composites showed that in the terminal region, *G*′ profoundly increases with the addition of Cu flakes, i.e., by ~six orders of magnitude, indicating geometrical entanglement of particles and the formation of a network. In the composites, the relative movement of particles is hindered by adjacent ones; therefore, the viscous response of the material is obstructed, which can be observed at the highest concentration of Cu flakes by increases in *G*″ and η* by ~four and ~five orders of magnitude, respectively. The same viscoelastic response may be observed for all the studied materials.

**Figure 4:**
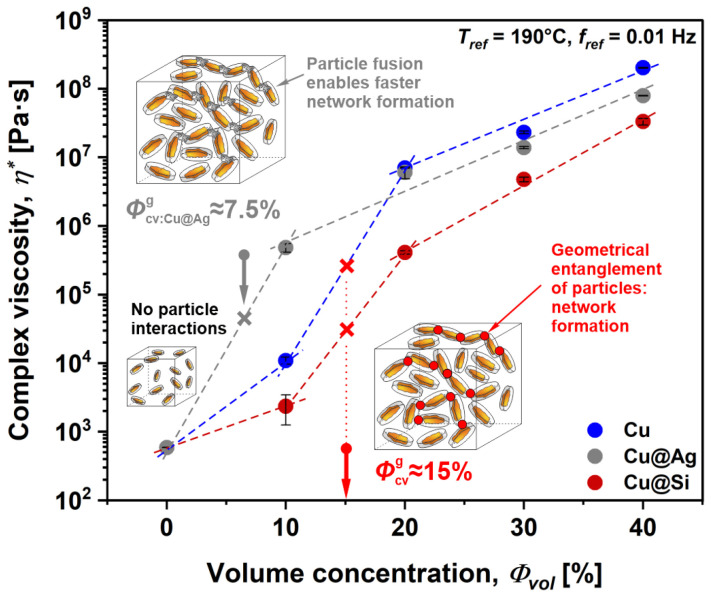
Complex viscosity as a function of volume concentration for Cu composites with different surface modifications.

The network formation of the investigated composites was determined by observing η* in the terminal region, here selected at 0.01 Hz, as a function of volume concentrations *Φ_vol_* (Figure 4). Results show a sigmoidal-shaped curve or S-curve in which the network formation was identified at an inflection point, representing the critical volume concentration at which Cu flakes geometrically entangle, which is also known as the geometrical percolation point, *Φ_cv_*_,*g*_ [34]. Below *Φ_cv_*_,*g*_, particles rotate freely about their center mass, mainly acting on the matrix material. However, above *Φ_cv_*_,*g*_ particles form a randomly connected network and exhibit long-range interactions, which profoundly affect the viscoelastic material response and functionality. Based on the analysis, *Φ_cv_*_,*g*_ was for the uncoated Cu and silica-coated Cu@Si composites determined to be ~15%, which is also within the usual value range reported in the literature for this particle geometry [35]. Surprisingly, the silver-coated Cu@Ag composites exhibited a much lower *Φ_cv_*_,*g*_, i.e., ~7.5%. Since *Φ_cv_*_,*g*_ is directly related to particle geometry, this decrease may be explained by an increasing particle aspect ratio *A_r_*, here related to the fusion of flakes, resulting from the thermo-chemical reaction of the Ag coating material [36,37]. These results could be extremely beneficial when evaluating the electrical functionality of Cu@Ag composites as the network is formed at lower *Φ_vol_*, while also exhibiting more pathways for electron transfer compared to the other investigated materials. However, once the network was established, all investigated composites exhibited similar viscoelastic responses, indicating that copper flakes display main load transfer capabilities regardless of their surface modification.

### 3.2. Thermal Analysis (Effect of Particle Interactions on Morphological Structure)

**Figure 5:**
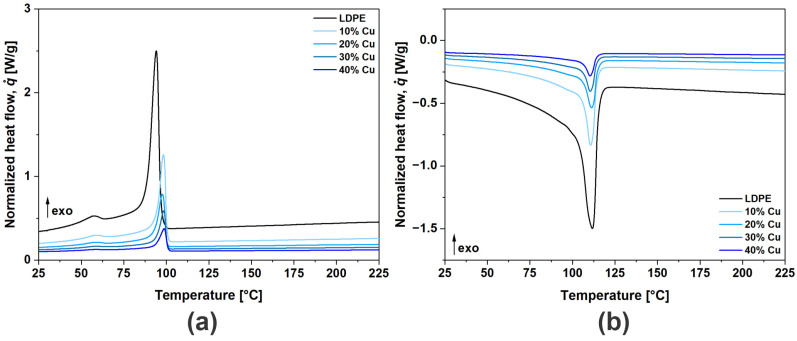
Thermograms: (**a**) cooling (crystallization kinetics); (**b**) 2nd heating (melting kinetics) for composites with uncoated Cu flakes.

When investigating conductive polymer composites, thermal analysis is commonly used as it enables insight into morphological (structural) changes of the matrix material, driven by particle interactions (network formation), as well as thermo-chemical or other reactions of the filler materials [38,39]. Those changes are usually determined by observing the crystallization and melting kinetics during cooling and 2nd heating, through so-called thermograms. For convenience, only thermograms for composites with uncoated Cu flakes are presented in Figure 5, however, the same trends were observed for the other two investigated materials, i.e., Cu@Ag and Cu@Si. The results show that the addition of Cu flakes affects the crystallization and melting kinetics, shifting peaks while reducing the thermal response, i.e., exo- and endothermic reactions, which will be subsequently addressed in more detail.

**Figure 6:**
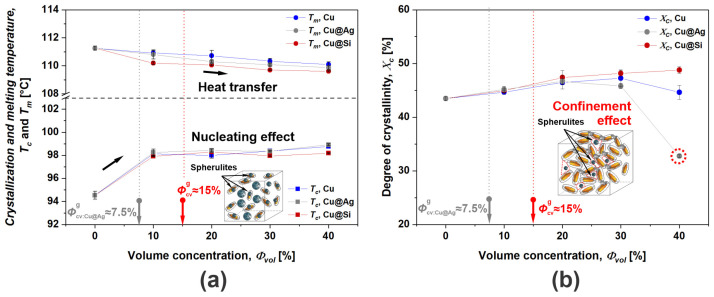
(**a**) Melting and crystallization temperatures and (**b**) degree of crystallinity as a function of volume concentration for the composites with and without surface modification of Cu flakes.

From the resulting thermograms, crystallization *T_c_* and melting *T_m_* temperatures with corresponding enthalpies Δ*H_c_* and Δ*H_m_* were identified. Based on the enthalpies, the degree of crystallinity *X_c_* (degree of molecular order in lamellas) was calculated, as *X_c_* affects the mechanical response of investigated composites [40]. From the results (Figure 6), it can be observed that the addition of Cu flakes affects *T_c_* and *T_m_*, whereas surface modifications provide only minor changes to those temperatures. The decrease in *T_m_* with volume concentration of added flakes was less than 2 °C regardless of surface modification and could be attributed to the thermal conductivity (heat transfer) of the flakes [41]. Conversely, *T_c_* instantly increased by ~4 °C at 10 vol.% of Cu flakes since the flakes reduce the thermal kinetics of the surrounding polymer molecules, hence acting as nucleating agents [39]. Further increases in flake concentration only slightly increased *T_c_*. The results of crystallinity analysis indicate that surface modification of Cu flakes notably influences the morphology at higher flake concentrations, while their amount plays a minor role. The slight increase in *X_c_* at lower concentrations is related to the nucleating effect; moreover, at the highest volume concentration, i.e., 40 vol.%, a further increase in *X_c_* was expected since the mold temperature had to be increased to enable sample manufacturing [42]. However, at the highest concentration of Cu@Ag filler, *X_c_* significantly decreased by ~10%. Considering the results of rheological analysis, this drop was attributed to the fusion of Cu@Ag flakes hindering crystal growth, which is also known as the confining effect [38,42]. This is unusual for LDPE, which has a branched molecular structure and could form lamellas elsewhere (on other branched or side chains). Furthermore, geometrical entanglement (network formation) of surface-modified Cu flakes represented by *Φ_cv_*_,*g*_ does not influence morphological changes as no inflection was observed in the *T_c_*, *T_m_* or *X_c_* curves, indicating weak links among particles inherited from the flake geometry.

### 3.3. Dynamic Mechanical Analysis (Effect of Particle Interactions on Mechanical Reinforcement)

**Figure 7:**
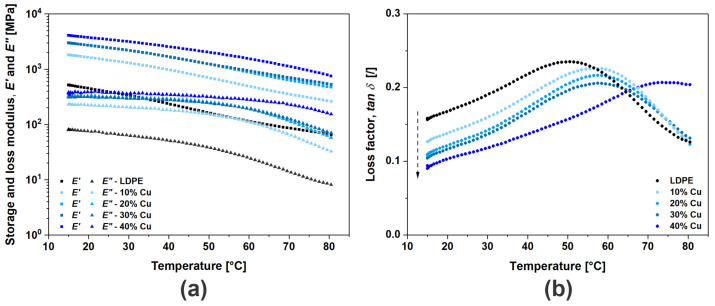
Viscoelastic properties (**a**) storage and loss modulus, (**b**) *tan δ* as a function of temperature for Cu composites.

Dynamical mechanical analysis, DMA, is a common characterization technique for investigating the mechanical response of polymers and polymer-based composites [43]. Since DMA is highly sensitive to polymer relaxation dynamics, phase transitions, morphology and other structural changes, it also offers the principal outlook on the mechanics of particle interactions as well as interfacial and other mechanisms (network formation, adhesion, etc.) when dealing with composites. The above-mentioned influences can be observed through the viscoelastic properties, i.e., storage modulus (*E*′) and loss modulus (*E*″). For convenience, only the viscoelastic properties for uncoated Cu composites are presented in Figure 7, which include the damping ratio *tan δ*, where phase shift *δ* represents the ratio of a material’s viscous contribution *E*″ to the elastic response *E*′. The same behavior was also observed for the composites with Cu@Ag and Cu@Si flakes.

Since the mobility of the molecules increases at higher temperatures, the results of temperature sweep tests (Figure 7) indicate a decrease in viscoelastic dynamic moduli (*G*′ and *G*″) with increasing temperature. However, the addition of Cu flakes not only significantly alters the viscoelastic behavior but also stabilizes the thermal response of the investigated composites. The increase in *E*′ with volume concentration *Φ_vol_* for ~3× (300%) is mainly related to the mechanics of load transfer from particles (flakes) to the matrix material. In this case, geometrically entangled Cu flakes hinder/obstruct the disentanglement of polymer molecules (reducing relative displacement), and thereby reinforce the material. Furthermore, the increase in *E*″ is less pronounced, i.e., ~one order of magnitude. Since *E*″ is proportional to the dissipated energy during the loading cycle, its increase is related to the internal friction (dissipative process) among entities, which increases as the number of flakes increases and reduces the overall free volume within the material. For this reason, the composites with higher *Φ_vol_* of flakes became stiffer, which can be observed by the decreasing *tan δ* with increasing *Φ_vol_* throughout the complete temperature range. However, the peak *tan δ*, here denoted as *tan δ_max_*, in relation to the α-relaxation temperature (transition of interfacial chains between crystalline and amorphous phases), which is reported to be ~70 °C [44,45], should be noted. The shift in *tan δ_max_* at the highest filler concentration to lower values (by ~0.1) and a higher temperature (by ~10 °C) indicates that the addition of Cu flakes enhances the adhesion among entities while obstructing the relaxation of interfacial chains, which the stabilizes thermo-mechanical response of the investigated composites. A similar trend was observed when Cu particles were added to acrylonitrile butadiene styrene (ABS) polymer [46].

**Figure 8:**
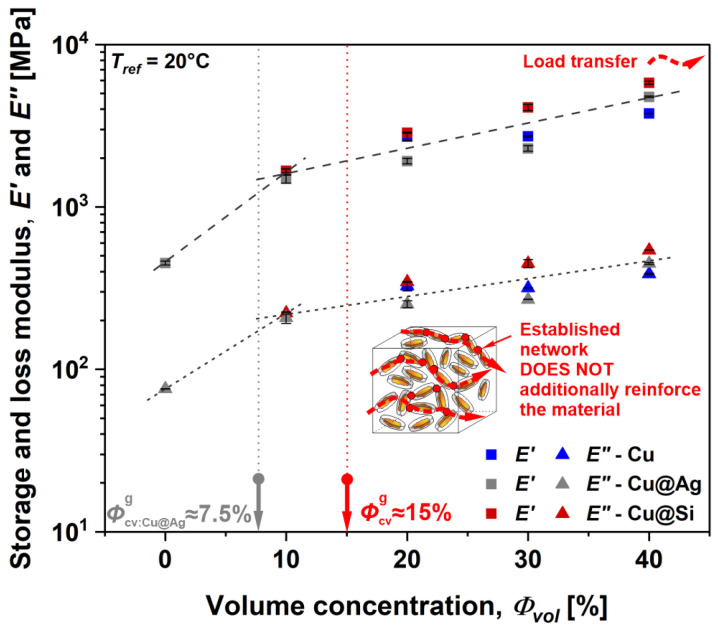
Viscoelastic properties as a function of volume concentration at 20 °C for the composites with and without surface modification of Cu flakes.

Furthermore, particle interactions and their contribution to mechanical reinforcement were evaluated by observing *E*′ and *E*″ as a function of *Φ_vol_* at 20 °C (Figure 8). In this case, a low temperature was selected due to the high elastic response of the material indicating low molecular mobility. As previously stated, dynamic moduli increase with *Φ_vol_* due to efficient load transfer from Cu flakes to the matrix material. However, the geometrical entanglement (network formation) represented by *Φ_cv_*_,*g*_ does not affect the mechanical response as no inflection of the curve was observed at the characteristic values. This indicates weak mechanical links between the particles inherited from the flake-type geometry. Furthermore, all investigated composites exhibited a similar magnitude of viscoelastic response, indicating that Cu flakes display main load transfer capabilities regardless of their surface modification.

### 3.4. Electrical Analysis (Effect of Particle Interactions on Electrical Conductivity)

**Figure 9:**
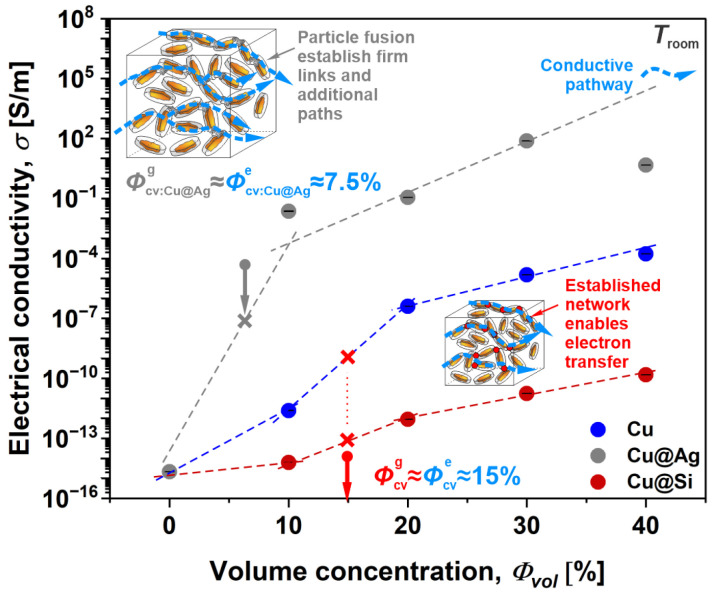
Electrical conductivity of Cu composites with different surface modifications.

The improvement in the electrical performance of the investigated materials with the addition of surface-modified Cu flakes was observed with 4-wire DC measurements, commonly applied when dealing with conductive polymer composites. Since all materials exhibit an ohmic response to voltage excitation, the electrical conductivity (*σ*) determined shows that the addition of Cu flakes as well as their surface modification significantly influences electrical properties (Figure 9). Moreover, it can be seen from the figure that the transition of polymer composites from insulator (LDPE polymer matrix) to conductor (addition of Cu-based particles) could be characterized by percolation theory. The application of percolation theory provides valuable insights into the critical volume concentration at which conductive pathways are established in the polymer composites, elucidating fundamental aspects of their electrical behavior.

From the obtained S-curve, the conductive network was identified with an inflection point, representing the critical volume concentration at which the Cu flakes establish conductive pathways. This point, where *σ* sharply increases with increasing *Φ_vol_*, is also known as the electrical percolation point, *Φ_cv_*_,*e*_. Based on the analysis, *Φ_cv_*_,*e*_ was determined to be ~15% for the uncoated Cu and silica-coated Cu@Si composites, while the Cu@Ag composite displayed an *Φ_cv_*_,*e*_ of ~7.5%. For the Cu and Cu@Si composites, similar values of *Φ_cv_*_,*e*_ were also reported in the literature [35]; however, the decrease in *Φ_cv_*_,*e*_ of the Cu@Ag composites was not expected. Comparable results for the establishment of an electrical percolation network in copper-based composites have also been reported in the literature. The studies confirm that the establishment of an electrical percolation network depends on the nature of the polymer matrix, as well as the particle size, shape and processing technique [5,47,48,49].

Nevertheless, the obtained results primarily indicate that geometrical entanglement is crucial for the establishment of conductive pathways within such composites, i.e., *Φ_cv_*_,*g*_ = *Φ_cv_*_,*e*_, as flakes do not inherit a particular alignment. Moreover, they also confirm the fusion of Cu@Ag flakes as a result of the thermo-chemical reaction of the Ag coating material [50], which was also observed through rheological and thermal analyses.

The results presented are profoundly beneficial in a view of the electrical performance of composites as the fusion of Cu@Ag flakes significantly reduces *Φ_cv_*_,*e*_, while exhibiting superior conductivity, i.e., *σ_Cu@Ag_* = 7.4 × 10^1^ S/m, compared to uncoated flakes, i.e., *σ_Cu_* = 1.7 × 10*^−^*^4^ S/m (~5 orders of magnitude lower) and silica-coated flakes, i.e., *σ_Cu@Si_* = 1.5 × 10*^−^*^10^ S/m (~11 orders of magnitude lower) composites. Such improvements are mainly related to the higher number of established conductive pathways, firm connections for electron transfer and the highly conductive and oxidation-resistant coating material. These findings have significant implications for the development of advanced materials for various applications, including flexible electronics, electromagnetic interference shielding and conductive adhesives, where precise control over electrical conductivity is paramount.

## 4. Conclusions

Copper flakes are excellent fillers for improving the electrical and mechanical properties of polymer-based composites. The presented study has shown that the network formation and surface modification of the Cu flakes significantly affect conductivity and reinforce the functionality of composites. Injection-molded polymer composites with three different commercially available fillers, consisting of uncoated Cu, Ag-coated (Cu@Ag) and Si-coated (Cu@Si) Cu flakes in low-density polyethylene–LDPE matrix material were prepared with volume concentrations of up to 40 vol.%.

The viscoelastic properties of the composite melts were determined by the oscillatory frequency sweep method at 190 °C. The concentration dependency of the complex viscosity (η*) at *f_ref_* = 0.01 Hz exhibited an S-shaped curve in which the establishment of a conductive network structure (geometrical percolation) was determined at the critical volume concentration *Φ_cv_*_,*g*_, at which Cu flakes geometrically entangle. Based on the analysis, *Φ_cv_*_,*g*_ depended on the surface modification of the flakes and was for uncoated Cu and silica-coated Cu@Si composites found to be ~15%, while, surprisingly, *Φ_cv_*_,*g*_ for silver-coated Cu@Ag composites was determined to be a much lower value, i.e., ~7.5%. Moreover, once the network was established, the viscoelastic response of all composites studied was similar, indicating that copper flakes display main load transfer capabilities regardless of their surface modification.

Thermal analysis showed that the presence of the Cu-based filler in the LDPE polymer changes the melting and crystallization properties. The highest change in *T_m_* and *T_c_* was observed at 10 vol.% filler, while negligible changes were observed with further increases in filler content. All composites exhibited a higher degree of crystallinity (*X_c_*) compared to neat LDPE, except for the composite with 40 vol.% Cu@Ag, where a significant decrease in the degree of crystallinity indicated the fusion of Cu@Ag flakes, which hindered crystal growth. Moreover, the geometrical entanglement (network formation) of surface-modified Cu flakes represented by *Φ_cv_*_,*g*_ did not show any influence on morphological changes as no inflection was observed in the *T_c_*, *T_m_* or *X_c_* curves, indicating weak links among particles inherited from the flake geometry.

The DMA characterization of solid-state mechanical properties showed that the presence of Cu-based particles contributed to the reinforcement of the LDPE matrix. Dynamic moduli increased with increasing *Φ_vol_* due to efficient load transfer from the Cu flakes to the matrix material; however, the geometrical entanglement (network formation) represented by *Φ_cv_*_,*g*_ did not affect the mechanical response as no inflection in the curve was observed at the characteristic values; again, indicating that Cu flakes display main load transfer capabilities regardless of their surface modification.

The most significant changes were observed when comparing the concentration-dependent electrical conductivity. The obtained S-curve enabled the determination of electrical network formation, represented by critical volume concentration, at which the Cu flakes establish conductive pathways. The results showed that critical volume concentrations of geometrical and electrical network formation were the same, indicating that geometrical entanglement is crucial for the establishment of conductive pathways within the studied composites as the flakes do not inherit a particular alignment. Moreover, the highest electrical conductivity was achieved in the Cu@Ag composites *(σ_Cu@aAg_* = 74.4 S/m) in which the fusion of Cu@Ag flakes significantly reduced *Φ_cv_*_,*e*_ and increased the conductivity.

The presented results have high applicative value in the production of reinforced and highly conductive Cu-based composites, where selective (conductive, rheological, mechanical) properties can be predicted by using copper fillers with metal-modified surfaces. Further research could be focused on the study of the long-term stability of Cu-based coated particles under various environmental conditions, as well as their performance in different polymer matrices.

## Data Availability

The original contributions presented in the study are included in the article, further inquiries can be directed to the corresponding author/s.
